# Characterization of a soybean (*Glycine max* L. Merr.) germplasm collection for root traits

**DOI:** 10.1371/journal.pone.0200463

**Published:** 2018-07-11

**Authors:** Harrison Gregory Fried, Sruthi Narayanan, Benjamin Fallen

**Affiliations:** 1 Department of Plant and Environmental Sciences, Clemson University, Clemson, South Carolina, United States of America; 2 Pee Dee Research and Education Center, Clemson University, Florence, South Carolina, United States of America; Estacion Experimental del Zaidin, SPAIN

## Abstract

Root systems that improve resource uptake and penetrate compacted soil (hardpan) are important for improving soybean (*Glycine max* L. Merr.) productivity in optimal and sub-optimal environments. The objectives of this research were to evaluate a soybean germplasm collection of 49 genotypes for root traits, determine whether root traits are related with plant height, shoot dry weight, chlorophyll index, and seed size, and identify genotypes that can penetrate a hardpan. Plants were maintained under optimal growth conditions in a greenhouse. Single plants were grown in mesocosms, constructed of two stacked columns (top and bottom columns had 25 and 46 cm height, respectively, and 15 cm inside diameter) with a 2-cm thick wax layer (synthetic hardpan; penetration resistance, 1.5 MPa at 30°C) in between. Plants were harvested at 42 days after planting. Significant genetic variability was observed for root traits in the soybean germplasm collection, and genotypes that penetrated the synthetic hardpan were identified. Genotypes NTCPR94-5157, NMS4-1-83, and N09-13128 were ranked high and PI 424007 and R01-581F were ranked low for most root traits. Shoot dry weight and chlorophyll index were positively related with total root length, surface area, and volume, and fine root length (Correlation coefficient, r ≥ 0.60 and P-value < 0.0001 for shoot dry weight and r ≥ 0.37 and P-value < 0.01 for chlorophyll index]. Plant height was negatively correlated with total root surface area, total root volume, and average root diameter (|r| ≥ 0.29, P-value < 0.05). Seed size was not correlated with any root traits. The genetic variability identified in this research for root traits and penetration are critical for soybean improvement programs in choosing genotypes with improved root characteristics to increase yield in stressful or optimum environments.

## Introduction

Soybean (*Glycine max* L. Merr.) is the fourth most important crop in the world in terms of area harvested and production [1]. Soybean is the most important oilseed and one of the most important and least expensive protein sources produced worldwide [2]. Soybean production is largely affected by several abiotic stresses, and drought is a major environmental factor limiting soybean yield worldwide and in the United States [3, 4]. Even though several soybean breeding programs in the country focus on drought tolerance, farmers still lack locally adapted, drought tolerant varieties, creating an urgent need for developing such varieties for improving soybean yields.

Productivity of any plant in optimal and suboptimal environment is often controlled by distribution and architecture of the root system [5, 6]. Carter [7] suggested that root systems that enhance soil water extraction would be the most promising target for improving soybean drought tolerance. However, the root, which is referred to as the “hidden half” of a plant [8], is challenging to study, major reasons being the phenotypic plasticity of roots in response to physical, chemical, and biological factors in the soil, lack of high-throughput and cost-effective screening methods, and difficulty to harvest roots from the soil without significant root loss [9, 10, 11].

Role of a root system in improving water and nutrient use efficiencies is well recognized in legume crops, including soybean [7, 12, 13, 14]. Genetic variability of root traits and its relationship with water and nutrient acquisition have been documented in legumes such as common bean (*Phaseolus vulgaris* L.) [15], chickpea (*Cicer arietinum* L.) [12] and lentil (*Lens culinaris* L.) [16]. Even though soybean breeders have taken significant efforts to introduce genetic variability in their populations, very limited research has been taken place to evaluate genetic variability for root traits in this crop. As a result, limited progress has been made in improving root system morphology and architecture of this crop that will increase resource acquisition. Exploring genetic variability of root traits will identify contrasting genotypes for root traits that can be included in crop improvement programs and help develop varieties with drought tolerance and/or resource capture. Determining the relationship of root traits with shoot and seed traits that are easily selectable such as plant height, shoot dry weight, chlorophyll index, and seed size will further improve utilization of root traits for crop improvement in optimal and suboptimal environments.

Soybean crop, in many instances, are grown on soils with a compacted zone or hardpan, worldwide. Most sandy soils in the coastal plains of the southeastern United States have an inherent hardpan. The hardpan limits root penetration, restricts root exploration and access to water and nutrients, and thus, reduces yields [17, 18, 19]. Additionally, soil hardpans make plants more susceptible to drought stress by reducing the extent to which plants can exploit stored soil water in deep horizons [20]. To manage soil compaction, farmers rely heavily on deep tillage, which is expensive in terms of time and energy and non-sustainable. In addition, the effects of deep tillage are temporary as the compacted layer forms again within a few years [21]. A viable alternative is to develop cultivars with root systems that penetrate the hardpan and alleviate compaction with minimum cost, maintaining sustainability. However, root penetrability has never been incorporated into soybean breeding programs for yield or drought tolerance, a major reason being the lack of information regarding genotypes that can penetrate a hardpan.

The objectives of this research were to evaluate a soybean germplasm collection of 49 genotypes for root traits, determine whether root traits have any relation with plant height, shoot dry weight, chlorophyll index, and seed size, and identify genotypes that can penetrate a hardpan.

## Materials and methods

### Germplasm

The germplasm used in this study consisted of 49 soybean genotypes including elite South Carolina breeding lines (n = 3); lines with exotic pedigree (n = 12); lines that have the ability to sustain nitrogen fixation under drought conditions (n = 3); genotypes having large and small seed sizes (n = 4 and 3, respectively); forage soybean (n = 2); check varieties (n = 4); slow wilting/pedigree tracing back to a slow wilting line (n = 7), fast wilting (n = 3), intermediate in wilting (n = 1), drought tolerant (n = 1), non-nodulating (n = 1), and moderately flood tolerant (n = 1) genotypes; a resistant cultivar to multiple races of soybean cyst nematode (n = 1); and wild soybean (*Glycine soja*) (n = 3) (Table 1). The soybean genotypes belonged to maturity groups IV, V, VI, VII, and VIII (n = 5, 8, 9, 18, and 9, respectively).

**Table 1:** Soybean genotypes used in the study, their maturity group, and characteristics.

No.	Genotype	Pedigree	Maturity group	Genus and Species	Characteristics/Comments	Source of information	Geographical Origin
1	LG11-3187	F6 Dwight (4) x PI 441001 (*Glycine tomentella*)	IV	*Glycine max*	Exotic pedigree	[22]	IL, United States
2	LG11-3370	F6 Dwight (4) x PI 441001 (*Glycine tomentella*)	IV	*Glycine max*	Exotic pedigree	[22]	IL, United States
3	LG11-4475	F2 Dwight (6) x PI 441001 (*Glycine tomentella*)	IV	*Glycine max*	Exotic pedigree	[22]	IL, United States
4	LG12-2271	F3:5 LG06-2340 x LG06-5920 (Derived from *Glycine tomentella*, PI 441001)	IV	*Glycine max*	Exotic pedigree	[23]	IL, United States
5	PI 549046	*Glycine soja*	IV	*Glycine soja*	Wild	[24]	Shaanxi, China
6	Essex	Lee x S5-7075	V	*Glycine max*	Fast wilting	Prior research of authors (unpublished data)	VA, United States
7	Osage	Hartz H5545 x KS4895	V	*Glycine max*	Moderately flood tolerant	[25]	AR, United States
8	PI 407191	*Glycine soja*	V	*Glycine soja*	Wild	[24]	Kyonggi, South Korea
9	PI 424007	*Glycine soja*	V	*Glycine soja*	Wild	[24]	Kyonggi, South Korea
10	R01-416F	Jackson x KS 4895	V	*Glycine max*	Sustained nitrogen fixation under drought	[26]	AR, United States
11	R01-581F	Jackson x KS 4895	V	*Glycine max*	Sustained nitrogen fixation under drought	[26]	AR, United States
12	R10-2436	R01-52F x R02-6268F	V	*Glycine max*	Sustained nitrogen fixation under drought	[27]	AR, United States
13	Vance	Essex x *Glycine soja*	V	*Glycine max*	Small seed size^†^	[28]	NC, United States
14	Boggs	G81-152 x Coker 6738	VI	*Glycine max*	Intermediate in wilting	[29]	GA, United States
15	N04-9646	BOGGS x NTCPR94-5157	VI	*Glycine max*	Slow wilting	[29]	NC, United States
16	N06-7023	N98-7265 x N98-7288	VI	*Glycine max*	Slow wilting	[30]	NC, United States
17	N07-14182	N7002 x Clifford	VI	*Glycine max*	Exotic pedigree	[31]	NC, United States
18	N10-7121	NC-Roy x 398833-BB	VI	*Glycine max*	Exotic pedigree	[30]	NC, United States
19	N11-9298	N03-12249 x N03-11895	VI	*Glycine max*	Exotic pedigree	[32]	NC, United States
20	NC-Roy	Holiday x Brim	VI	*Glycine max*	Fast wilting	[29]	NC, United States
21	Nitrasoy	D68-099 x Cook	VI	*Glycine max*	Non-nodulating	[24]	NC, United States
22	TC11ED-90	N6202 x AGS-363	VI	*Glycine max*	Large seed size^‡^	Diversity Yield Trials^§^ in 2013	NC, United States
23	Benning	Hutcheson x Coker 6738	VII	*Glycine max*	Fast wilting	[33]	GA, United States
24	G00-3213	N7001 x Boggs	VII	*Glycine max*	Check^¶^	[30, 34]	GA, United States
25	Gasoy 17	Bragg x Hood	VII	*Glycine max*	Drought tolerant	Personal Communication	GA, United States
26	N06-7543	NC Roy x N8001	VII	*Glycine max*	Pedigree traces back to a slow wilting line, PI 471938	[35]	NC, United States
27	N09-12854	N7103 x PI408337-BB	VII	*Glycine max*	Exotic pedigree	[32]	NC, United States
28	N09-13128	N7002 x Tamahakari-BB	VII	*Glycine max*	Exotic pedigree	[30]	NC, United States
29	N09-13890	TCPR-83 x 11136	VII	*Glycine max*	Slow wilting (Pedigree traces back to a slow wilting line, PI 471938)	[35] Prior research of authors (unpublished data)	NC, United States
30	N10-7320	11936 x Boggs	VII	*Glycine max*	Slow wilting (Pedigree traces back to a moderately slow wilting line PI 471931)	Prior research of authors (unpublished data)	NC, United States
31	N7001	N77-114 x PI416937	VII	*Glycine max*	Check	[36]	NC, United States
32	N7003CN	Cook x Anand	VII	*Glycine max*	Resistant to multiple races of Soybean Cyst Nematode	[37]	NC, United States
33	N7103	NTCPR90 x Pearl	VII	*Glycine max*	Small seed size	[38]	NC, United States
34	NC-Raleigh	N85-492 x N88-480	VII	*Glycine max*	Check	[39]	NC, United States
35	NMS4-1-83	N7103 x PI 366122 (*Glycine soja*)	VII	*Glycine max*	Exotic pedigree	[30]	NC, United States
36	NTCPR94-5157	Davis x N73-1102	VII	*Glycine max*	Slow wilting	[29]	NC, United States
37	Santee	Coker 82–622 x Hutcheson	VII	*Glycine max*	Check	[40]	SC, United States
38	SC-14-1127	NC Raleigh x PI 378696B (*Glycine soja*)	VII	*Glycine max*	Exotic pedigree	[24]	SC, United States
39	TC11ED-28	N6202 x AGS-363	VII	*Glycine max*	Large seed size	Diversity Yield Trials in 2015	NC, United States
40	TCWN05/06-5068	Cook x SC97-1821	VII	*Glycine max*	Large seed size	[41]	NC, United States
41	Crockett	PI 171451 x Hampton 266	VIII	*Glycine max*	Forage	[24, 42]	TX, United States
42	Jing Huang 18	Unknown	VIII	*Glycine max*	Forage	[24]	Hubei, China
43	N05-7432	N7002 x N98-7265	VIII	*Glycine max*	Slow wilting	[43]	NC, United States
44	N09-13671	N98-7961 x N02-8718	VIII	*Glycine max*	Exotic pedigree	[30]	NC, United States
45	N8101	NC114 x N7101	VIII	*Glycine max*	Small seed size	[28]	NC, United States
46	NLM09-52	N6202 x G98SF114.	VIII	*Glycine max*	Large seed size	[32]	NC, United States
47	SC06-291RR	SC98-1930 x SC00-892RR	VIII	*Glycine max*	Elite South Carolina breeding line^#^	N/A	SC, United States
48	SC07-1518RR	SC01-809RR x G99-3211	VIII	*Glycine max*	Elite South Carolina breeding line	N/A	SC, United States
49	SC10-394RR	SC98-2070 x SC01-783RR	VIII	*Glycine max*	Elite South Carolina breeding line	N/A	SC, United States

^†^Individual seed weight ≤ 0.09 g.

^‡^Individual seed weight ≥ 0.20 g.

^§^Southern Collaborative Soybean Diversity Yield Trials MG VII-VIII supported by the United Soybean Board

^¶^Soybean lines with high yields in the Southeast, and which are used in regional breeding trials as benchmarks with which yield of other lines are compared. They were developed in SC, NC, or GA, and have been thoroughly tested under multiple environments on multiple soil types for several years.

^#^Current lines in the South Carolina breeding program with high yields in the recent years

### Experimental details

**Fig 1:**
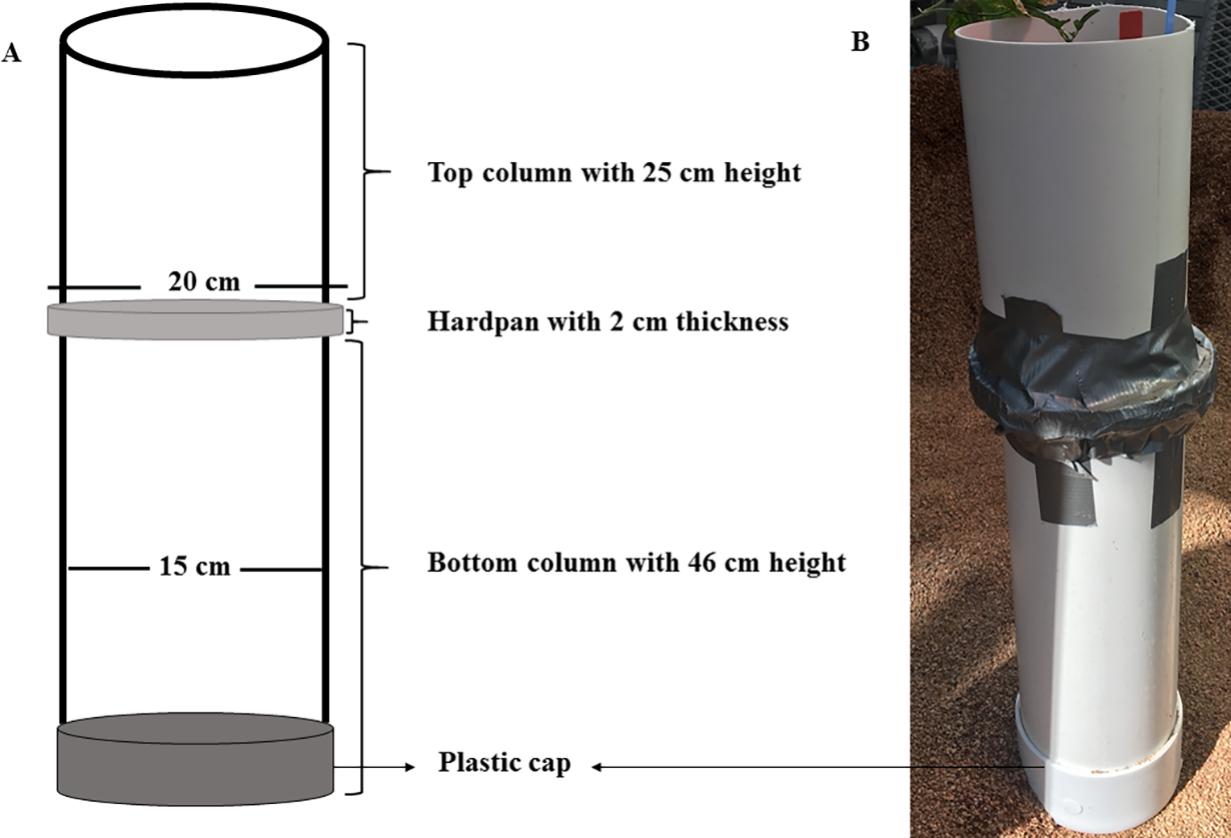
The mesocosm used to grow soybean plants in the experiment. Diagram of a mesocosm that was constructed of two stacked polyvinyl chloride (PVC) columns with an inside diameter of 15 cm (A). The height of the bottom and top columns were 46 and 25 cm, respectively. Each mesocosm was sealed at the bottom with a plastic cap, which had a central hole of 0.5 cm diameter for drainage. The synthetic hardpan made up of paraffin wax and petroleum jelly placed in between the top and bottom columns had a diameter of 20 cm and thickness of 2 cm. A photograph of the mesocosm (B). The top and bottom columns along with the synthetic hardpan in between were tightly sealed together with a duct tape as shown in Fig 1B.

This research was conducted under controlled environmental conditions in a greenhouse at the Department of Plant and Environmental Sciences, Clemson University, Clemson, SC. Two independent experiments (Run 1 and 2) were conducted to examine the variability of root traits in the soybean germplasm collection of 49 genotypes. The soybean plants were grown in mesocosms constructed of two stacked polyvinyl chloride (PVC) columns with an inside diameter of 15 cm (Fig 1). The height of the bottom and top columns were 46 and 25 cm, respectively. Each mesocosm was sealed at the bottom with a plastic cap, which had a central hole of 0.5 cm diameter for drainage. The bottom column was filled with saturated Turface MVP (Burnett Athletics, Campobello, SC). Turface is calcined, non-swelling illite and silica clay. Turface was chosen as the rooting medium as it allows for easy separation of roots, relative to traditional soil and potting mixture [44, 45]. In order to measure the root penetration ability of compacted rooting medium, a synthetic hardpan made up of paraffin wax and petroleum jelly was placed on top of the bottom column. The use of a wax-petroleum jelly system has been shown to be a suitable method for studying root penetration in several field crops [19, 46, 47, 48, 49, 50, 51, 52]. A major advantage of this system is that, unlike in the case of compacted soil layers, the changes in water content does not affect physical properties of the wax and petroleum jelly [19]. The wax- petroleum jelly hardpans used in this study consisted of 85% wax (Royal Oak Enterprises LLC, Roswell, GA) and 15% petroleum jelly (Vaseline; Unilever, Englewood Cliffs, NJ) by weight, and had a strength (penetration resistance) of 1.5 MPa at 30°C (S1 Fig). The mixture was melted at 80°C, poured into molds, and allowed to solidify at room temperature. The resulting wax- petroleum jelly disks had a diameter of 20 cm and thickness of 2 cm. The top column was placed on top of the wax-petroleum jelly synthetic hardpan. In this way, the synthetic hardpan was imposed at 25 cm depth in each mesocosm. The top and bottom columns along with the synthetic hardpan (slightly larger diameter than the columns) in between were tightly sealed together with a duct tape that prevented roots from circumventing the synthetic hardpan. After that, the top column was filled with saturated turface as the rooting medium. The turface in the top column was fertilized with a controlled-release fertilizer, Osmocote with 18:6:12, N:P_2_O_5_:K_2_O (Scotts, Marysville, OH) at a rate of 20 g per column before sowing. A systemic insecticide, Marathon (a.i.: Imidacloprid: 1–[(6–Chloro–3–pyridinyl)methyl]–N–nitro–2–imidazolidinimine; OHP, Inc., Mainland, PA) was also applied to the top column at a rate of 1.7 g per column before sowing to control sucking pests, such as aphids (*Aphis glycines* Matsumura), thrips [*Neohydatothrips variabilis* (Beach) and *Frankliniella spp*.], and whiteflies (*Bemisia tabaci*). Ten seeds of each genotype were weighed to estimate seed size (individual seed weight). Three seeds of a single genotype were sown in each column at a depth of 4 cm. Sowing occurred on 9 September 2016 for run 1 and 20 February 2017 for run 2. After emergence, only the healthiest plant out of the three was retained in each column, and the other two were removed. Plants were watered every 10 days at approximately 10 ml per column and maintained under optimum temperature conditions (30/20°C, daytime maximum/nighttime minimum) [53] and at a photoperiod of 13 hours until harvest [54]. Plants were harvested at 42 days after sowing. Eighty and 25% of the plants reached flowering stage in run 1 and 2, respectively at the time of harvest. No pest problems were observed on the plants in both runs.

### Data collection

Plant height and chlorophyll index were measured at the time of harvest. Plant height was determined as the distance from the base of the plant to the tip of the top trifoliate [55]. Chlorophyll index was measured using a self-calibrating chlorophyll meter (Soil Plant Analyzer Development (SPAD), Model 502 Plus; Spectrum Technologies, Plainfield, IL, USA). Measurements were taken at six different areas on the top trifoliate (two measurements on each of the three leaflets), and the readings were averaged to get a single value for a plant. At harvest, plants were cut at the base to separate shoots from the roots. Shoots were packed in paper bags and dried to constant weight at 60°C for determining dry weight. Before harvesting roots, the duct tape that sealed the top and bottom columns with a hardpan in between, was removed. After that, each mesocosm was gently inverted at about 140°C to let the contents (turface with the root system and the hardpan) slip down to the ground. Roots from the top and bottom columns and the hardpan were harvested separately. Roots were separated from the turface carefully to eliminate root loss and breakage. The hardpans were carefully broken apart to measure root penetration, which was defined as the depth of the hardpan to which the roots penetrated, where maximum and minimum penetrations were 2 cm and 0 cm, respectively. After harvest, root system of each plant was washed, placed between wet paper towels, sealed in Ziploc bags (S.C. Johnson & Sons, Inc. Racine, WI), and stored at 4°C (roots from the top and bottom columns and the hardpan were washed, packed, and stored separately for any plant that penetrated the hardpan). For further root analysis, roots from the top and bottom columns and the hardpan were scanned separately using an Epson Perfection V600 scanner (6400 dpi resolution) (Epson, Long Beach, CA). To prepare root samples for scanning, the roots were taken out of the Ziploc bags and submerged in water within a tray (25 cm x 20 cm x 2 cm). This was to maximize separation and minimize overlap of roots. The root systems were scanned while submerged in water in the tray. The scanned images of roots were analyzed using WinRHIZO Pro image analysis system (Regent Instruments, Inc., Quebec City, QC) to estimate total root length (sum of the lengths of all roots in the root system), total root surface area, total root volume, average root diameter, and fine root (diameter <0.25mm) length and surface area. For those plants, which root systems penetrated the hardpan, the root data from the top and bottom columns and the hardpan were combined for data analysis (i.e., the total or fine root length, surface area, and volume for a root system was the sum of those measures in the top and bottom columns and the hardpan. Root diameter values in the top and bottom columns and the hardpan were averaged to estimate the average root diameter of the root system).

### Statistical analyses

The experimental design was a randomized complete block with four replications in both runs. Analysis of variance was performed on genotypes using the GLIMMIX procedure in SAS (Version 9.4, SAS Institute) for root and shoot traits. The probability threshold level (α) was 0.05. Genotype was treated as a fixed effect and replication nested within run was treated as a random effect. Run, replication, and genotype were the class variables. Separation of means was done using the LSD test (P<0.05). The CORR and REG procedures in SAS were used to find the relationships among root and shoot traits. Principal component analysis was carried out using the PRINCOMP procedures in SAS on root and shoot traits of all genotypes. A biplot was generated using the JMP software.

## Results

### Genetic variability of root traits

**Fig 2:**
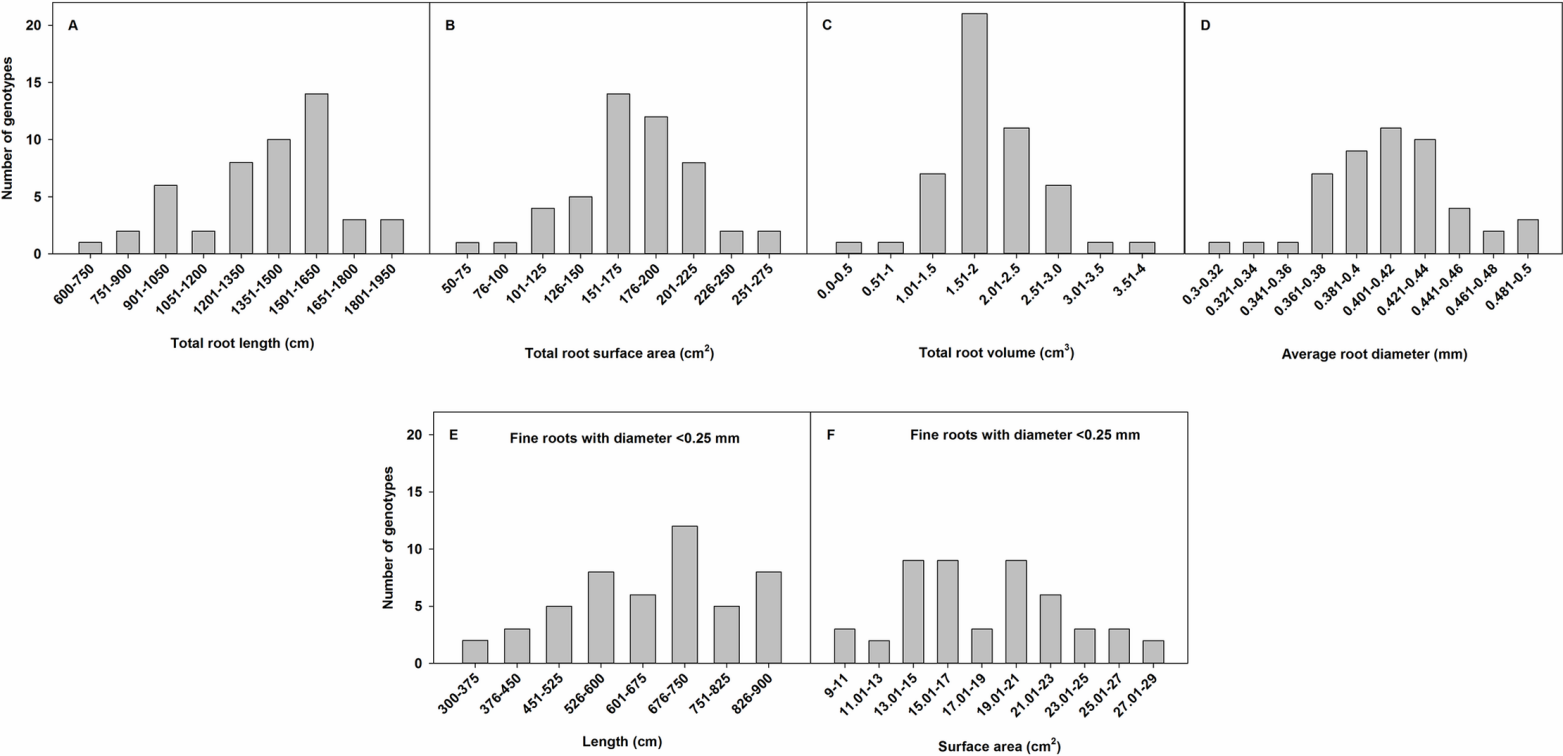
Distribution of total root length, total root surface area, total root volume, average root diameter, fine root (diameter < 0.25 mm) length, and fine root surface area among 49 soybean genotypes. The y-axis indicates the absolute number of genotypes in each root trait class.

Significant variability was observed for root traits among the soybean genotypes (Table 2). Because there was no significant interaction between run and genotype for all root traits except penetration, data were combined across runs for the root traits, except penetration. Data were analyzed separately for each run for penetration. A wide range was observed for all root traits with more than 150% variation between minimum and maximum values of all traits except average diameter (53%) (Table 2). Frequency distributions of root traits (Fig 2) showed the extent of genetic variability for these traits. Root traits followed a normal distribution (P > 0.05, Shapiro–Wilk test) (Fig 2). Six and 12% of the genotypes were included in the lower and upper extreme classes (600–900 cm and 1651–1950 cm, respectively) of total root length; similarly, 4 (50–100 cm^2^) and 8% (226–275 cm^2^) for total root surface area, 4% each (0–1 cm^3^ and 3.01–4.0 cm^3^) for total root volume, 4 (0.30–0.34 mm) and 10% (0.461–0.50 mm) for average root diameter, 10 (300–450 cm) and 27% (751–900 cm) for fine root length, and 10% each (9–13 cm^2^ and 25.01–29 cm^2^) for fine root surface area (Fig 2).

**Table 2:** Analysis of variance results on effects of run (the study was conducted two times, which were designated as two runs), rep(run), genotype, and run x genotype interaction and range for various root traits.

Trait	P values				Range	Coefficient of variation^‡^ (%)
	Run	Rep(run)	Genotype	Run x Genotype		
Total root length (cm)	0.0005	0.3652	0.0003	0.4541	646–1949	21
Total root surface area (cm^2^)	0.4021	0.3181	0.0011	0.2864	59–271	24
Total root volume (cm^3^)	0.1318	0.3933	0.0349	0.3110	0.45–3.52	31
Average root diameter (mm)	0.0032	0.0702	0.3074	0.6598	0.32–0.49	9
Penetration^†^ (cm)	0.1713	0.6253	0.5034	< .0001	0.00–1.50 (Run 1) 0.00–0.28 (Run 2)	390 (Run 1) 396 (Run 2)
**Traits of fine roots with diameter < 0.25 mm**						
Length (cm)	0.0002	0.3315	0.1116	0.7551	355–900	23
Surface area (cm^2^)	< .0001	0.6809	0.2405	0.5015	9.17–27.28	26

^†^Root penetration of a synthetic hardpan (2 cm thickness) that simulate a compacted soil layer

^‡^Ratio of the standard deviation to the mean (average)

Eighteen genotypes penetrated the hardpan fully or partially in at least one run (Table 3). Among them, four were slow wilting/having pedigree tracing back to a slow wilting line (NTCPR94-5157, N09-13890, N06-7543, and N06-7023), four were of exotic pedigree (N07-14182, N10-7121, LG12-2271, and LG11-4475), three were of large seed size (NLM09-52, TCWN05/06-5068, and TC11ED-28), and two were check varieties (NC-Raleigh and N7001). The other five included fast wilting (Benning) and moderately flood tolerant (Osage) cultivars, a genotype with small seed size (N8101), one that sustains nitrogen fixation under drought (R10-2436), and a forage soybean cultivar (Crockett). Six of the 18 genotypes that penetrated the hardpan (at least partially) were released cultivars (Benning, Osage, NC-Raleigh, N7001, N8101, and Crockett). The slow wilting line NTCPR94-5157 was the only genotype that penetrated the hardpan completely in at least one run. Genotypes NC-Raleigh, N06-7023, N09-13890, LG12-2271, Benning, and Crockett penetrated the hardpan in both runs. Interestingly, none of the elite South Carolina breeding lines and *G*. *soja* lines penetrated the hardpan in either runs.

**Table 3:** Soybean root penetration of synthetic hardpans (2 cm thickness) that simulate compacted soil layers. Penetration was defined as the depth of the synthetic hardpan to which the roots penetrated, where maximum and minimum penetrations are 2 cm and 0 cm, respectively. Genotypes that penetrated the hardpan in at least one run are given below.

Genotype	Penetration (cm)	
	Run 1	Run 2
NTCPR94-5157	2.00±0.30^a^^†^	0
N10-7121	0.50±0.26^b^	0
NC-Raleigh	0.67±0.30^b^	0.08±0.14^a^
Crockett	0.40±0.26^b^	0.05±0.14^a^
Benning	0.15±0.26^b^	0.25±0.14^a^
LG12-2271	0.30±0.26^b^	0.13±0.14^a^
TCWN05/06-5068	0.25±0.26^b^	0
N06-7023	0.05±0.26^b^	0.25±0.14^a^
N07-14182	0.15±0.26^b^	0
N09-13890	0.10±0.26^b^	0.17±0.17^a^
R10-2436	0.13±0.26^b^	0
N7001	0.05±0.26^b^	0
Osage	0	0.13±0.14^a^
N8101	0	0.09±0.14^a^
LG11-4475	0	0.15±0.14^a^
N06-7543	0	0.08±0.17^a^
TC11ED-28	0	0.09±0.14^a^
NLM09-52	0	0.28±0.14^a^

^†^Mean ± standard error. Values followed by different letters are significantly different according to a LSD test at P<0.05.

The genotypes were ranked according to the numerical values of the root traits (Table 4). Genotype NTCPR94-5157 (slow wilting) had the highest total root length and total root surface area. This genotype was also ranked as one among the top three for total root volume, fine root length, and fine root surface area. Similarly, genotype NMS4-1-83 (exotic pedigree) was ranked as one among the top three for total root length, total root surface area, total root volume, fine root length, and fine root surface area, and as one among the top five for average root diameter. Another genotype with exotic pedigree, N09-13128, was ranked as one among the top 10 for total root length, total root surface area, total root volume, fine root length, and fine root surface area. In addition, genotypes N07-14182, N7003CN, Essex, Santee, LG11-4475, TCWN05/06-5068, G00-3213, N09-13671, Jing Huang 18, and N10-7121 were included in the top 10 for most (at least three) root traits.

**Table 4:** Soybean genotypes that were ranked high and low for total root length, total root surface area, total root volume, average root diameter, and fine root (diameter <0.25 mm) length and surface area.

	Total root length (cm)	Total root surface area (cm^2^)	Total root volume (cm^3^)	Average root diameter (mm)	Traits of fine roots with diameter < 0.25 mm	
					Length (cm)	Surface area (cm^2^)
Highest 10^†^	NTCPR94-5157 (1949±237)^‡^	NTCPR94-5157 (270.57±26.32)	NMS4-1-83 (3.52±0.45)	LG12-2271 (0.49±0.05)	N09-13128 (900±205)	G00-3213 (27.28±17.80)
	NMS4-1-83 (1860±229)	NMS4-1-83 (270.31±24.70)	NTCPR94-5157 (3.16±0.47)	N06-7543 (0.49±0.05)	NMS4-1-83 (894±205)	NMS4-1-83 (27.15±17.80)
	N09-13128 (1802±229)	N07-14182 (240.95±24.70)	Jing Huang 18 (2.84±0.47)	Jing Huang 18 (0.48±0.05)	NTCPR94-5157 (875±209)	NTCPR94-5157 (26.65±17.86)
	N07-14182 (1755±229)	N09-13128 (227±24.70)	LG11-4475 (2.80±0.45)	N05-7432 (0.47±0.05)	TCWN05/06-5068 (867±205)	TCWN05/06-5068 (26.35±17.80)
	N7003CN (1741±247)	N09-13671 (224.55±26.32)	N07-14182 (2.78±0.45)	NMS4-1-83 (0.47±0.05)	Essex (850±205)	SC06-291RR (25.15±17.86)
	Essex (1702±229)	Jing Huang 18 (223.61±26.32)	N09-13671 (2.60±0.47)	N7001 (0.46±0.05)	G00-3213 (849±205)	N09-13128 (24.85±17.80)
	Santee (1633±237)	LG11-4475 (222.65±24.70)	N10-7121 (2.58±0.45)	N10-7121 (0.45±0.05)	Santee (834±209)	Santee (23.60±17.86)
	LG11-4475 (1619±229)	N10-7121 (222.07±24.70)	LG12-2271 (2.55±0.45)	LG11-4475 (0.45±0.05)	Nitrasoy (826±209)	N7103 (23.28±17.80)
	TCWN05/06-5068 (1610±229)	N7003CN (216.08±28.30)	N09-13128 (2.40±0.45)	N09-13671 (0.44±0.05)	N7003CN (820±214)	Essex (22.84±17.80)
	G00-3213 (1600±229)	LG11-3370 (207.59±31.09)	NC-Roy (2.26±0.45)	TC11ED-90 (0.44±0.07)	N7103 (793±205)	SC10-394RR (22.62±17.80)
Lowest 10	PI 424007 (646±237)	PI 424007 (59.48±26.32)	PI 424007 (0.45±0.47)	PI 424007 (0.32±0.05)	N06-7543 (355±214)	N06-7543 (9.17±17.94)
	PI 549046 (877±247)	N09-12854 (100.34±26.32)	N09-12854 (0.98±0.47)	Nitrasoy (0.34±0.05)	TC11ED-90 (371±247)	R01-581F (10.59±18.23)
	R01-581F (875±279)	Boggs (105.45±24.70)	SC-14-1127 (1.11±0.50)	N11-9298 (0.36±0.05)	PI 549046 (380±214)	LG12-2271 (10.63±17.80)
	N09-12854 (902±237)	R01-581F (113.08±34.40)	R01-581F (1.14±0.60)	N09-12854 (0.36±0.05)	R01-581F (398±231)	TC11ED-90 (11.33±18.51)
	Boggs (919±229)	SC-14-1127 (113.38±28.37)	Boggs (1.19±0.47)	R01-581F (0.37±0.06)	PI 424007 (399±209)	PI 549046 (12.85±17.94)
	N06-7543 (930±247)	PI 549046 (115.46±28.30)	PI 549046 (1.23±0.50)	Boggs (0.37±0.05)	N05-7432 (454±205)	PI 407191 (13.05±17.80)
	SC-14-1127 (964±247)	Crockett (135.69±24.70)	Nitrasoy (1.29±0.47)	Essex (0.37±0.05)	LG12-2271 (495±205)	N09-13671 (13.20±17.86)
	TC11ED-90 (1002±308)	SC07-1518RR (141.34±24.70)	Crockett (1.38±0.45)	NLM09-52 (0.37±0.05)	Boggs (503±205)	Gasoy 17 (13.56±17.80)
	N05-7432 (1014±229)	Nitrasoy (146.07±26.32)	SC07-1518RR (1.44±0.45)	R01-416F (0.37±0.05)	N09-12854 (512±209)	N05-7432 (13.70±17.80)
	Crockett (1123±229)	TC11ED-90 (148.57±39.61)	N11-9298 (1.52±0.45)	N04-9646 (0.38±0.07)	SC-14-1127 (514±214)	PI 424007 (13.73±17.86)
LSD	492	75	1.29	0.1	317	12.18

^†^Genotypes were ranked based on the numerical values of root traits.

^‡^Values in parentheses are means ± standard errors of the respective traits.

Genotype PI 424007 (*G*. *soja*; wild) had the lowest total root length, total root surface area, total root volume, and average root diameter, compared to all other soybean genotypes (Table 4). This genotype was also ranked as one among the lowest 10 for fine root length and fine root surface area. Genotype R01-581F (sustained nitrogen fixation under drought conditions) was ranked as one among the lowest 10 for total root length, total root surface area, total root volume, average root diameter, fine root length, and fine root surface area. In addition, genotypes PI 549046, N09-12854, Boggs, N06-7543, SC-14-1127, TC11ED-90, N05-7432, Crockett, R01-416F, and Nitrasoy were included in the bottom 10 for most (at least three) root traits.

**Fig 3:**
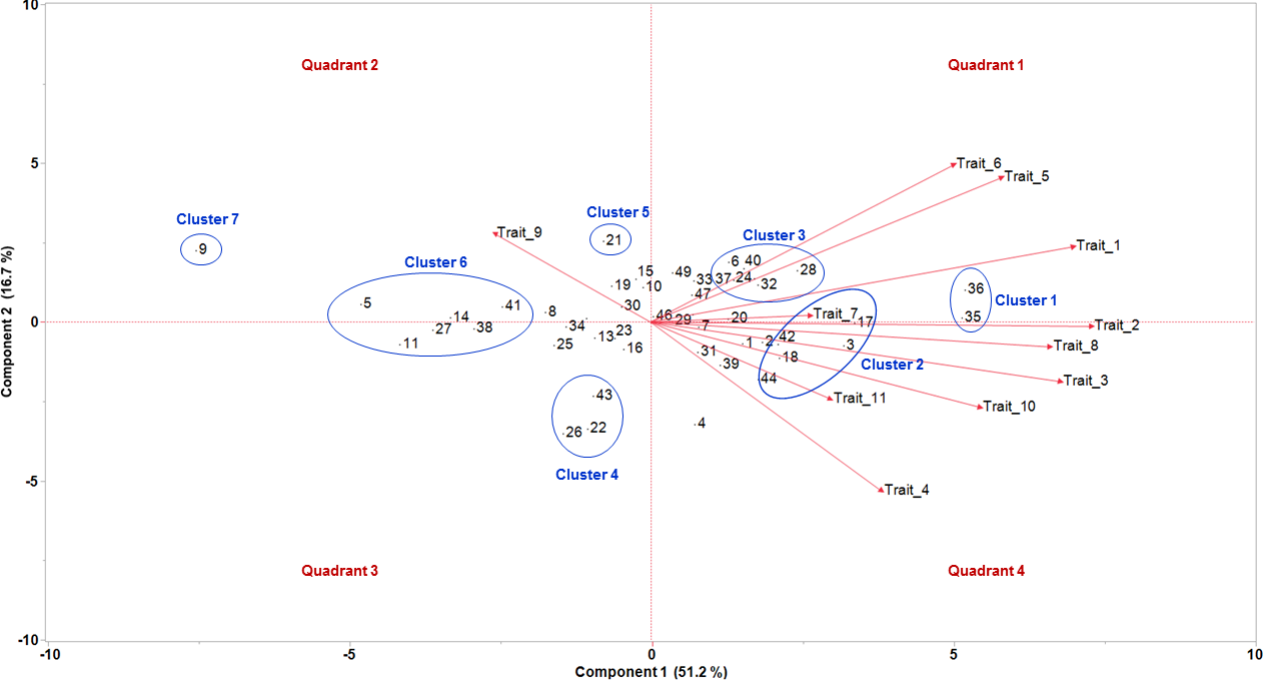
Principal component analysis biplot that separated the soybean genotypes in to clusters based on the root and shoot traits. Traits 1–11 are total root length, total root surface area, total root volume, average root diameter, fine root (diameter < 0.25 mm) length, fine root (diameter < 0.25 mm) surface area, root penetration, shoot dry weight, plant height, chlorophyll index, and seed size, respectively. Genotypes 1–49 are marked on the biplot; please see Table 1 for the genotype names corresponding to the numbers.

We conducted a principal component analysis (PCA) based on all phenotypic data and generated a biplot to investigate the possibility of clustering of genotypes (Fig 3). The biplot separated the genotypes in to seven clusters. Cluster 1 included genotypes NTCPR94-5157 and NMS4-1-83, which were ranked among the top three for most root traits. Cluster 2 (genotypes N07-14182, LG11-4475, N09-13671, Jing Huang 18, and N10-7121) and cluster 3 (genotypes N09-13128, N7003CN, Essex, Santee, TCWN05/06-5068, and G00-3213) included other genotypes that were ranked among the top 10 for at least three root traits. Genotype PI 424007, which had the lowest total root length, total root surface area, total root volume, and average root diameter, was clearly separated from all other genotypes (Cluster 7). Cluster 4 (genotypes N05-7432, TC11ED-90, and N06-7543), Cluster 5 (genotype Nitrasoy), and Cluster 6 (genotypes PI 549046, R01-581F, N09-12854, SC-14-1127, Boggs, and Crockett) included genotypes that were ranked among the bottom 10 for at least three root traits. All genotypes that were ranked among the top 10 for at least three root traits (Clusters 1, 2, and 3) were included in the quadrants 1 and 4, whereas, all genotypes that were ranked among the bottom 10 for at least three root traits (Clusters 4, 5, 6, and 7) were included in the quadrants 2 and 3. The most important root traits contributing to the clustering pattern were total root surface area, total root length, total root volume, fine root length, and fine root surface area.

### Relations among root and shoot traits

Shoot dry weight was positively related with total root length, total root surface area, total root volume, fine root length, and fine root surface area (Pearson correlation coefficient, r ≥ 0.45) (Table 5). Particularly, the relations of shoot dry weight with total root length, total root surface area, and total root volume were strong with r ≥ 0.79 (Table 5, S2 Fig). Chlorophyll index was positively related with total root length, total root surface area, total root volume, and fine root length (r ≥ 0.37) (Table 5, S2 Fig). Plant height was not related with total root length, fine root length, and fine root surface area, and was negatively correlated with total root surface area (r, -0.29), total root volume (r, -0.34), and average root diameter (r = -0.29) (Table 5, S2 Fig). Seed size did not have any significant relation with total root length, total root surface area, total root volume, average root diameter, fine root length, and fine root surface area (Table 5).

**Table 5:** Correlations among various root and shoot traits of the 49 soybean genotypes.

	Total root surface area	Total root volume	Average root diameter	Fine root (diameter < 0.25 mm) length	Fine root (diameter < 0.25 mm) surface area	Shoot dry weight	Plant height	Chlorophyll index	Seed size
Total root length	0.93^†^***^‡^	0.77***	NS^§^	0.92***	0.79***	0.79***	NS	0.55***	NS
Total root surface area		0.95***	0.58***	0.73***	0.60***	0.84***	-0.29*	0.65***	NS
Total root volume			0.76***	0.52***	0.42**	0.79***	-0.34*	0.64***	NS
Average root diameter				NS	NS	0.48**	-0.29*	0.51**	NS
Fine root (diameter < 0.25 mm) length					0.93***	0.60***	NS	0.37**	NS
Fine root (diameter < 0.25 mm) surface area						0.45**	NS	NS	NS
Shoot dry weight							NS	0.69***	0.43**
Plant height								-0.30*	-0.33*
Chlorophyll index									0.49**

^†^Values in each cell represent Pearson correlation coefficient.

^‡^*, **, and *** indicate significance at 0.05, 0.01, and 0.001, respectively

^§^Not significant at 0.05 probability level

Fine root traits were positively correlated with whole root system traits (Table 5). For example, fine root length had a strong positive correlation with total root length (r = 0.92, P-value <0.0001). Similarly, fine root surface area was strongly related with total root length (r = 0.79, P-value <0.0001). In addition, fine root length and surface area were positively related with total root surface area (r = 0.73, P-value <0.0001 and r = 0.60, P-value <0.0001, respectively) and volume (r = 0.52, P-value <0.0001 and r = 0.42, P-value = 0.003, respectively).

## Discussion

Considerable variability was detected for root traits in the soybean germplasm collection of 49 genotypes evaluated in this study. These genotypes were selected based on a variety of traits that are important for soybean improvement (e.g., slow wilting, nitrogen fixation under drought, and exotic pedigree, see Table 1). The variability of root traits we identified among the 49 genotypes is promising and warrants additional research to further explore the genetic diversity in wild and domesticated soybean. The methodology used in this study to estimate root penetration ability and other root traits could be used to identify soybean varieties that could be grown in arid regions and/or regions susceptible to the occurrence of hardpans.

The extent of variability for root traits among the soybean genotypes is demonstrated by the wide range observed for these traits (Table 2). The 49 soybean genotypes evaluated in this study belonged to maturity groups IV, V, VI, VII, and VIII. However, maturity groups did not influence any root traits [P-values for the effect of maturity groups on total root length, total root surface area, total root volume, average root diameter, fine root length, and fine root surface area were 0.72, 0.54, 0.35, 0.06, 0.74, and 0.51, respectively, and for root penetration, 0.19 (Run 1) and 0.89 (Run 2)]. Similar observations were made by Turman et al. [56], who observed that root length density (total root length in unit soil volume) of soybean was not related to maturity groups under field conditions.

This study evaluated root penetration ability of soybean genotypes using wax-petroleum jelly discs, which simulate compacted soil layers or soil hardpans. Analysis of variance detected significant interaction between run and genotype for root penetration (Table 2), and we analyzed the penetration data separately for each run (Table 3). Temperature influences the penetration resistance of the wax- petroleum jelly hardpans (S1 Fig). The differences in weather conditions during Run 1 and 2 might have influenced the greenhouse temperature slightly, which in turn influenced the penetration resistance of the hardpans. This might be the reason for differences in root penetration of genotypes between runs.

To the best of our knowledge, this study is the first one evaluating a diverse soybean germplasm collection for root penetration. Soil compaction occurs in nearly every farm in the United States, limiting root penetration and crop yields. In the southeastern United States, most soils have an inherent compacted layer of subsoil (hardpan), which often necessitates expensive and non-sustainable tillage operations to increase the rooting zone. Our study has identified soybean genotypes that penetrated the synthetic hardpans (Table 3). We found that eighteen genotypes penetrated the hardpan fully or partially in at least one run, and the behavior was consistent in both runs for six of them (NC-Raleigh, N06-7023, N09-13890, LG12-2271, Benning, and Crockett). These genotypes offer useful genetic material for breeders to develop high yielding soybean varieties for hardpan forming soils.

We have presented 10 genotypes that were ranked high and 10 genotypes that were ranked low for total root length, surface area, and volume, average root diameter, and fine root length and surface area in Table 4. These genotypes can be exploited to identify the genes or loci controlling the root traits and to improve drought tolerance and/or resource capture in soybean. Genotypes NTCPR94-5157, NMS4-1-83, and N09-13128 were ranked high and genotypes PI 424007 and R01-581F were ranked low for total root length, surface area, and volume and fine root length and surface area. The top performing genotype NTCPR94-5157 was a slow wilting genotype. ‘Slow wilting’ is a trait that is widely been used in the United States soybean breeding programs for developing drought tolerant varieties [57]. Although the physiological basis for slow wilting is not yet determined, it likely involves root traits that improve water use efficiency or water conservation during soil drying [58]. Thus, it could be reasoned that the increased length, surface area, and volume of the whole root system and the fine roots contribute to the slow wilting ability of the genotype NTCPR94-5157. Compared to all other genotypes, it had the largest penetration value in run 1 (200% higher than the second largest penetration value; Table 3). In addition to NTCPR94-5157, three other genotypes (N09-13890, N06-7543, and N06-7023) that penetrated the hardpan in both runs were slow wilting genotypes/having pedigree tracing back to a slow wilting line. The slow wilting nature of these genotypes combined with their ability to penetrate the hardpans makes them valuable genetic materials for breeding for drought tolerance in hardpan forming soils like that exists in the Southeastern United States.

In our study, we found that the fine root traits were related with the whole root system traits (Table 5). For example, fine root length and surface area were positively related with total root length, surface area, and volume with ‘r’ ranging between 0.42 and 0.92. Similar observations are reported by Prince et al. [59] who reported that fine root length, surface area, and volume had strong positive correlations with total root volume in soybean. Fine roots increase root surface area per unit mass [60]. Since they are the most active part of the root system in extracting water and nutrients [61, 62, 63], the enhanced resource capture achieved through fine roots might have increased total root length, surface area, and volume as well.

In the present research, shoot dry weight and chlorophyll index were positively correlated with total root length, total root surface area, total root volume, and fine root length (Table 5, S2 Fig). Shoot dry weight and chlorophyll index are easily selectable traits, and are commonly utilized by soybean improvement programs to select desired genotypes. Since selecting genotypes based on root traits is highly challenging in a soybean breeding program, the positive correlations of shoot dry weight and chlorophyll index with root traits are advantageous as the genotypes selected based on these easily measurable shoot traits can have improved root systems as well. Water and nutrient uptake from the soil is proportional to the contact area between root surface and soil [64]. This indicates that resource uptake increases with root surface area. Liang et al. [14] reported that total root length and surface area influence foraging and accumulation of nutrients such as phosphorus. Hudak and Patterson [65] found that a large root system, influenced by root length, surface area, and volume, enables the plant to exploit substantial soil volume, and is crucial for improving yield under drought conditions in soybean. In the present study, the increased resource capture achieved through larger root systems that were realized by increased root length, surface area, and volume might have contributed to increased dry matter addition, and thus, shoot dry weight. Additionally, better nitrogen uptake achieved through larger root systems might have contributed to increased chlorophyll index. On the other hand, the increased amount of photoassimilates as a result of increased leaf greenness (measured through chlorophyll index) and shoot growth might have been utilized to increase root growth. Taken together, our results suggest that chlorophyll index and shoot weight have the potential to be indirect selection criteria for root traits that contribute to high yield potential.

The absence of correlation between plant height and total root length and the negative correlations of plant height with total root surface area and total root volume do not support the view that selecting for decreased plant height can result in a small root system. These results are supported by our own previous research along with that of others on multiple crops including chickpea [66], field pea (*Pisum sativum* L.) [67], and wheat [44, 45, 68]. Total root length is determined by number and length of lateral roots [67], and is primarily controlled by different sets of genes, compared to plant height [68]. The negative correlations of plant height with total root surface area and total root volume may be because assimilates that are not used to increase plant height might have diverted to root system to add more surface area, and thus, volume. Contrasting reports exist in terms of correlation of seed size with root traits [44, 69, 70]. Seed size was not correlated with any root traits evaluated in the present research (Table 5). This shows that large seeds may not always produce long roots or large root systems.

In the United States, soybean breeders have pursued the promising approach of introducing exotic germplasm to their breeding programs to increase genetic diversity. This approach has been found to be useful for improving yield and drought tolerance [57, 58, 71]. Twelve soybean lines with exotic pedigree, which were included in the South Carolina breeding program, were tested in the present study for root traits. Six of them, NMS4-1-83 (N7103 x PI 366122), N09-13128 (N7002 x Tamahakari-BB), N07-14182 (N7002 x Clifford), N10-7121 (NC-Roy x 398833-BB), LG11-4475 (F2 Dwight (6) x PI 441001), and N09-13671 (N98-7961 x N02-8718) were ranked in the top 10 for most (at least three) root traits (Table 4).

*G*. *soja*, the putative ancestor of cultivated soybean (*G*. *max*), intercrosses easily with soybean, and has been utilized as an important resource for enhancing genetic diversity in soybean breeding populations [72, 73, 74]. The soybean germplasm tested in this study included three *G*. *soja* genotypes. Two of them (PI 549046 and PI 424007) were ranked in the lowest 10 for most (at least three) root traits (Table 4). Our results are supported by previous reports that root and shoot growth of *G*. *soja* are much lower than *G*. *max*, with *G*. *soja* producing thinner roots, reduced root mass, root volume, and narrow root hairs [59, 75]. This variability should be considered when making interspecific hybridizations in breeding programs. Interestingly, genotype NMS4-1-8, which was ranked as one among the top three for total root length, total root surface area, total root volume, fine root length, and fine root surface area, and as one among the top five for average root diameter, had *G*. *soja* (PI 366122) as one of its parents. Similarly, genotypes LG11-4475 and LG12-2271, which had *G*. *tomentella* (wild and perennial species of *Glycine*) in their parentage possessed improved root traits, including hardpan penetration.

## Conclusions

Significant genetic variability was observed for root traits in the soybean germplasm collection of 49 genotypes that was examined. Genotypes NTCPR94-5157 (slow wilting), NMS4-1-83 (exotic pedigree), and N09-13128 (exotic pedigree) were ranked high and genotypes PI 424007 (wild) and R01-581F (sustained nitrogen fixation under drought conditions) were ranked low for most root traits. Among them, genotype NTCPR94-5157 penetrated the hardpan in at least one run. To our best knowledge, the present study is the first one evaluating a diverse soybean germplasm collection for root penetration. The genotypes that were able to penetrate the synthetic hardpan offer useful genetic material for breeding programs to improve yield in hardpan forming soils like that exists in the Southeastern United States. We also examined whether root traits were related with plant height, shoot dry weight, chlorophyll index, and seed size, and found that only shoot dry weight and chlorophyll index were positively related with root traits, plant height was not correlated or had negative correlations with root traits, and seed size was not related with any root traits. The genetic variability identified in this research for root traits and penetration are critical for soybean improvement programs in choosing genotypes with improved root characteristics in order to improve drought tolerance and/or resource capture. The methodology used in this study to estimate root traits could be used to select soybean varieties that could be grown in arid regions and/or regions with hardpan forming soils.

## Supporting information

S1 Fig Strength (penetration resistance) of wax-petroleum jelly mixture as a function of temperature. The mixture was made of 85% paraffin wax and 15% petroleum jelly (Vaseline, Unilever, Englewood Cliffs, NJ) by weight. Wax and petroleum jelly were heated together to 80°C until both were completely melted and mixed together. The mixture was poured into mason jars until the jars were 3/4^th^ full. The wax and petroleum jelly mixtures in the mason jars were equilibrated to four different temperatures, 21, 25, 27, and 30°C, and the strength of the mixtures were measured as the resistance to penetration of a cone penetrometer (FieldScout SC900 Soil Compaction meter, Spectrum Technologies, Inc., Plainfield, IL). There were five replicated jars at each temperature.
(TIF)

S2 Fig Relation of total root length, surface area, and volume with shoot dry weight, chlorophyll index, and plant height of soybean genotypes. (TIF)

S1 File Excel file containing all data on root, shoot, and seed traits. (XLSX)
