# Supplementary figures and images for: Characterization of a soybean (Glycine max L. Merr.) germplasm collection for root traits
Source: PLoS One. 2018 Jul 11;13(7):e0200463. doi: 10.1371/journal.pone.0200463 (PMC6040769; doi:10.1371/journal.pone.0200463)

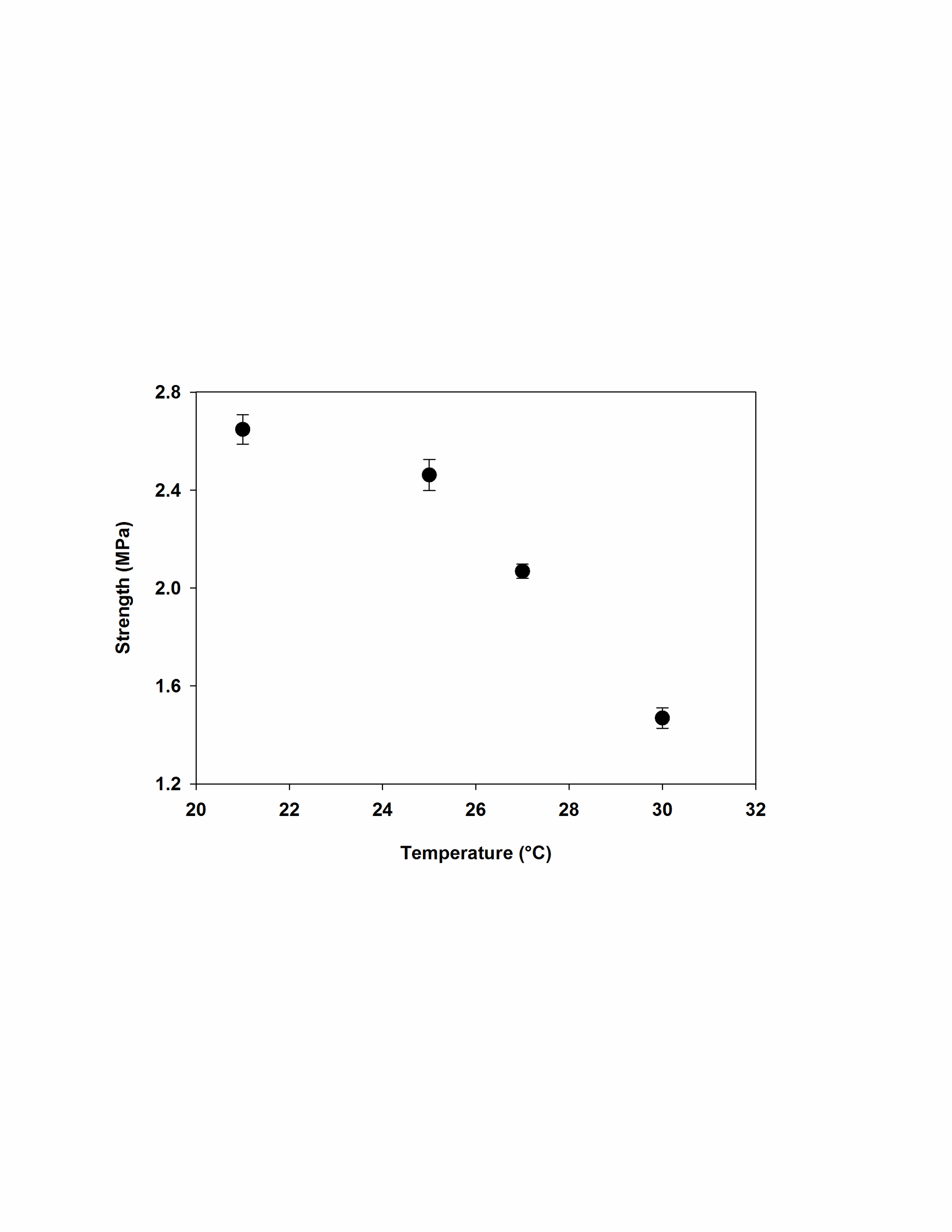

Supplement: S1 Fig — The mixture was made of 85% paraffin wax and 15% petroleum jelly (Vaseline, Unilever, Englewood Cliffs, NJ) by weight. Wax and petroleum jelly were heated together to 80°C until both were completely melted and mixed together. The mixture was poured into mason jars until the jars were 3/4th full. The wax and petroleum jelly mixtures in the mason jars were equilibrated to four different temperatures, 21, 25, 27, and 30°C, and the strength of the mixtures were measured as the resistance to penetration of a cone penetrometer (FieldScout SC900 Soil Compaction meter, Spectrum Technologies, Inc., Plainfield, IL). There were five replicated jars at each temperature. (TIF) [file pone.0200463.s001.tif]

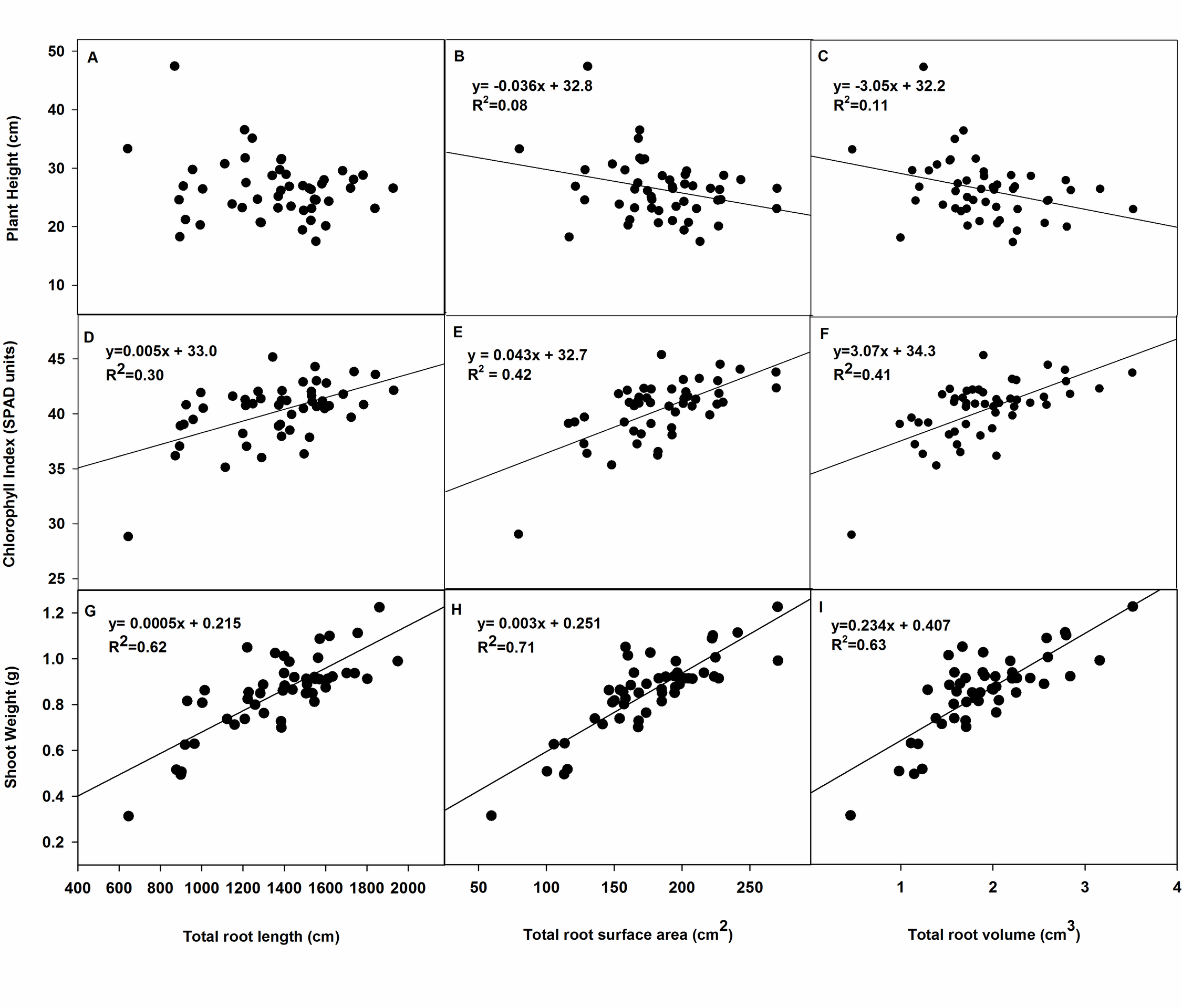

Supplement: S2 Fig — (TIF) [file pone.0200463.s002.tif]
